# Prostate cancer multiparametric magnetic resonance imaging visibility is a tumor-intrinsic phenomena

**DOI:** 10.1186/s13045-022-01268-6

**Published:** 2022-05-03

**Authors:** Amanda Khoo, Lydia Y. Liu, Taylor Y. Sadun, Amirali Salmasi, Aydin Pooli, Ely Felker, Kathleen E. Houlahan, Vladimir Ignatchenko, Steven S. Raman, Anthony E. Sisk, Robert E. Reiter, Paul C. Boutros, Thomas Kislinger

**Affiliations:** 1 Department of Medical Biophysics, University of Toronto, Toronto, ON Canada; 2 Princess Margaret Cancer Centre, University Health Network, Toronto, ON Canada; 3 Vector Institute, Toronto, ON Canada; 4 Department of Human Genetics, University of California, Los Angeles, Los Angeles, CA USA; 5 Jonsson Comprehensive Cancer Center, David Geffen School of Medicine, University of California, Los Angeles, Los Angeles, CA USA; 6 Department of Urology, David Geffen School of Medicine, University of California, Los Angeles, Los Angeles, CA USA; 7 Department of Radiology, David Geffen School of Medicine, University of California, Los Angeles, Los Angeles, CA USA; 8 Department of Pathology, David Geffen School of Medicine, University of California, Los Angeles, Los Angeles, CA USA; 9 Department of Pharmacology and Toxicology, University of Toronto, Toronto, ON Canada; 10 Institute for Precision Health, University of California, Los Angeles, Los Angeles, CA USA

**Keywords:** Proteomics, Multiparametric magnetic resonance imaging, Prostate cancer

## Abstract

Multiparametric magnetic resonance imaging (mpMRI) is an emerging standard for diagnosing and prognosing prostate cancer, but ~ 20% of clinically significant tumors are invisible to mpMRI, as defined by the Prostate Imaging Reporting and Data System version 2 (PI-RADSv2) score of one or two. To understand the biological underpinnings of tumor visibility on mpMRI, we examined the proteomes of forty clinically significant tumors (*i.e.*, International Society of Urological Pathology (ISUP) Grade Group 2)—twenty mpMRI-visible and twenty mpMRI-invisible, with matched histologically normal prostate. Normal prostate tissue was indistinguishable between patients with visible and invisible tumors, and invisible tumors closely resembled the normal prostate. These data indicate that mpMRI-visibility arises when tumor evolution leads to large-magnitude proteomic divergences from histologically normal prostate.

**Supplementary Information:**

The online version contains supplementary material available at 10.1186/s13045-022-01268-6.

## To the Editor,

Multiparametric magnetic resonance imaging (mpMRI) has dramatically enhanced the management of localized prostate cancer, providing an opportunity to improve diagnosis and risk stratification while simultaneously reducing unnecessary and risky needle biopsies [1]. However, because ~ 20% of clinically significant tumors remain invisible to mpMRI [2], there is limited consensus on when a biopsy can be safely avoided upon a negative mpMRI. The reasons for prostate cancer mpMRI invisibility are largely unknown, despite mpMRI-visible tumors harboring more adverse pathological and biological features [3–6]. Within International Society of Urological Pathology (ISUP) Grade Group 2, mpMRI visibility is associated with increased genomic instability, presence of intraductal carcinoma and/or cribriform architecture (IDC/CA) histology and hypoxia, a constellation of features termed *nimbosus *[3, 7]. Given the role of cellular density and perfusion in mpMRI, differences in stromal organization in non-malignant tissue [4] are hypothesized to affect water diffusion and thus mediate tumor microenvironmental influence on mpMRI visibility.

**Fig. 1:**
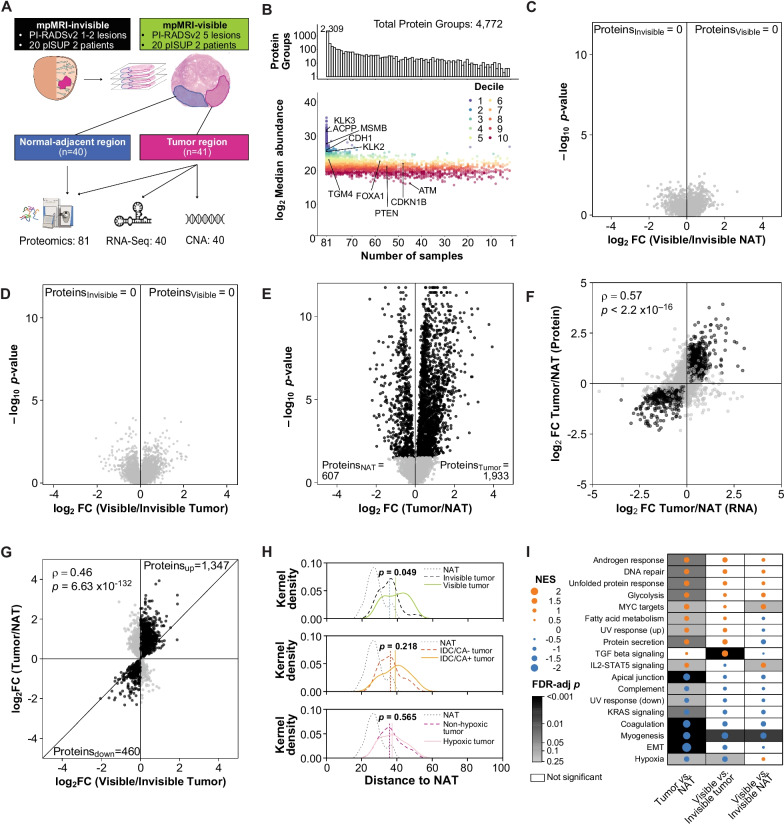
Proteomics of mpMRI visibility. **A** Sample outline. **B** Summary of quantified proteins in various number of samples. Differentially abundant proteins in mpMRI-visible (*n* = 20) and mpMRI-invisible (*n* = 20) NATs (**C**), mpMRI-visible (*n* = 21) and mpMRI-invisible tumors (*n* = 20) (**D**), and tumor (*n* = 40) and NAT regions (*n* = 40) (**E**). Statistically significant (FDR < 0.05, Mann–Whitney *U* test) proteins colored in black. **F** Comparison of tumor/NAT in the proteome (*n*_tumor_ = 40, *n*_NAT_ = 40) and transcriptome (*n*_tumor_ = 499, *n*_NAT_ = 53). Genes that were significantly associated with tumors or NATs at both the protein and RNA levels (FDR < 0.05) are colored in black. **G** Associations of protein abundance changes between tumor *versus* NAT and mpMRI-visible tumor *versus* mpMRI-invisible tumor, using proteins that were significantly differentially expressed in tumor *versus* NAT (*n* = 2540). Significant (FDR < 0.05) proteins from the tumor/NAT comparison that had the same directionality in the mpMRI-visible/invisible tumor comparison are colored in black. **H** Distribution of Euclidean distance between each group and median protein abundance in NATs. Only proteins that were quantified in all tumor and NAT samples were used (*n* = 2309). IDC/CA groups were determined based on the presence of intraductal carcinoma (IDC) or cribriform architecture (CA) histology (IDC/CA+, *n* = 11) or not (IDC/CA−, *n* = 29). Hypoxia groups (*n* = 20 per group) were determined by median dichotomization (median Ragnum score = −1). **I** Gene set enrichment analysis for 3 sets of comparisons (Tumor/NAT, mpMRI-visible/invisible tumor, and mpMRI-visible/invisible NAT) using the Hallmark gene set. The union of significant terms (FDR < 0.25) are shown. The size of the dot represents the magnitude of the effect, the color denotes the direction (positive: orange; negative: blue), and background shading the FDR-adjusted *p*-value. Only significant associations (FDR < 0.25) have gray background. mpMRI: multiparametric magnetic resonance imaging; PI-RADSv2: Prostate Imaging Reporting and Data System version 2; pISUP: pathological International Society of Urological Pathology Grade Group; CNA: Copy number abberation; NAT: normal tissue adjacent to the tumor; FDR: Benjamini–Hochberg false discovery rate; FC: fold change; *ρ*: Spearman’s rho; *p*: *p*-value; NES: normalized enrichment score; and IDC/CA: Intraductal carcinoma or cribriform architecture

To understand the biological underpinnings of tumor visibility on mpMRI, we performed global proteomics on twenty mpMRI-invisible (Prostate Imaging Reporting and Data System version 2 [PI-RADSv2] 1–2) and twenty mpMRI-visible (PI-RADSv2 5) tumors, all from patients with a solitary pathological ISUP Grade Group 2 lesion larger than 1.5 cm [3]. We analyzed both tumor and adjacent histologically normal tissue (NAT) from all patients, leading to 81 proteomes (Fig. 1A, Additional file 3: Table S1). A detailed description of the methods can be found in Additional file 1: Methods (available online).

We quantified 4772 proteins (Additional file 4: Table S2), of which 2309 were detected in all 81 samples (Fig. 1B). Clustering by protein abundance yielded four protein subtypes and four sample subtypes (Additional file 2: Fig. S1A). The sample subtypes were driven by differences between tumors and NATs (Adjusted Rand Index [ARI] = 0.22, *p* = 0.001) and not mpMRI visibility (ARI = − 0.01, *p* = 0.64). The protein subtypes reflected specific biological pathways. For example, P1 genes were associated with immune response and extracellular matrix organization and were more abundant in tumors than NATs (Additional file 5: Table S3).

To test the important and widespread hypothesis that the tumor microenvironment influences visibility on mpMRI [3, 8], we compared protein abundances between NATs from patients with mpMRI-visible and mpMRI-invisible tumors. To our surprise, not a single protein differed between the two groups (Fig. 1C). Similarly, differences in the proteomes of mpMRI-visible and mpMRI-invisible tumors were also small and not statistically significant, albeit with larger effect sizes compared to the result from NATs (Fig. 1D). In contrast, we observed the expected large, statistically significant differences between the proteomes of tumors and NATs (Fig. 1E). Similarly, large differences were observed at the transcriptome level (Additional file 1: Methods, Additional file 2: Fig. S1B), where most tumor/NAT proteomic differences were corroborated (Spearman’s *ρ* = 0.57, *p* < 2.2 × 10^–16^, Fig. 1F).

Given these modest differences between mpMRI-visible and mpMRI-invisible tumor proteomes, we hypothesized that mpMRI-invisible tumors might reflect an intermediate state between NATs and mpMRI visibility. Consistent with this, protein abundance differences associated with tumor mpMRI visibility were correlated with NAT-tumor differences (Spearman’s *ρ* = 0.46, *p* < 1 × 10^–16^, Fig. 1G). These associations were diminished in the NAT proteomes (Spearman’s *ρ* = 0.13, *p* = 7.01 × 10^–11^, Additional file 2: Fig. S1C), and in the matched tumor transcriptomes [3] (Spearman’s *ρ* = 0.00, *p* = 0.79, Additional file 2: Fig. S1D). The proteome of mpMRI-invisible tumors was more similar to that of NATs compared to the proteome of mpMRI-visible tumors (Fig. 1H), likely contributing to their invisibility. Consistently, normoxic tumors and tumors lacking IDC/CA histology were more similar to NATs (Fig. 1H). Altered pathways in mpMRI-visible tumors *vs.* mpMRI-invisible tumors overlapped substantially with those distinguishing tumors from NATs (hypergeometric test *p* = 5.5 × 10^–14^, Fig. 1I). Epithelial-to-mesenchymal transition and myogenesis genes were enriched in mpMRI-invisible tumors compared to mpMRI-visible tumors, consistent with reports that stromal and extracellular matrix genes were enriched in mpMRI-invisible tumors [4]. mpMRI-visible tumors were enriched in pathways associated with advanced disease, including androgen response, DNA repair, and MYC and TGF-β signaling [9]. Taken together, these data help explain the aggressive clinical behavior of mpMRI-visible tumors, concordant with increased *PTEN* loss [10], higher Oncotype and Decipher genomic classifier scores [5], and elevated nimbosus hallmarks [3].

**Fig. 2:**
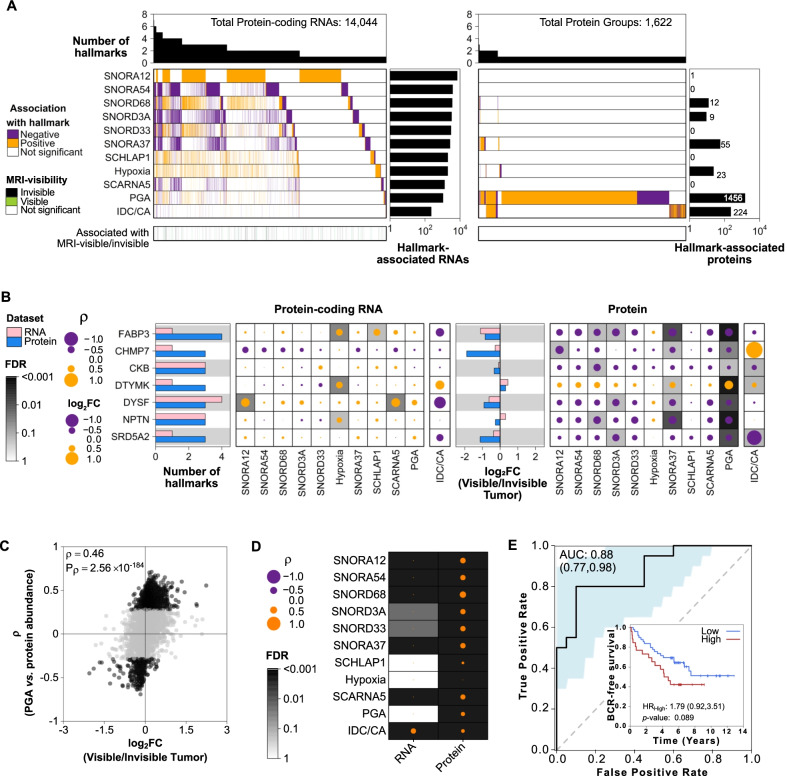
Protein associations with genomic, transcriptomic, and pathological hallmarks of mpMRI visibility. **A** Protein-coding RNAs (left) and proteins (right) associated with hallmarks of mpMRI visibility, colored by positive (orange) or negative (purple) associations. Top barplot shows the number of hallmarks each RNA or protein was associated with. Side barplot shows the number of validated RNAs or proteins associated with each hallmark (Additional file 1: Methods). Bottom covariate bar indicates significant RNAs or proteins associated with visible (green) or invisible (black) tumors (FDR < 0.05). **B** Genes that were associated with three or more hallmarks or mpMRI visibility at the protein level. Left barplot shows the number of hallmarks each gene is associated with at the RNA (pink) or protein (blue) level. Dot maps show the effect size of the association between gene expression and each hallmark. The size of the dot represents the magnitude of the effect, the color denotes the direction (positive: orange; negative: purple), and background shading the FDR. Only significant associations have a gray background. Right barplot shows the log_2_ fold change between mpMRI-visible and invisible tumors for RNA and protein. **C** Spearman’s correlation between tumor protein/PGA and protein/mpMRI visibility associations. Validated proteins with abundance significantly correlated with PGA (FDR < 0.2) are colored in black. **D** Summary of the correlation between associations with each hallmark and mpMRI visibility in protein-coding RNAs and proteins. **E** A 3-protein model classified mpMRI-visible tumors with an area under the curve (AUC) of 88%. AUC confidence intervals in parentheses and shaded in blue. Inset: The protein signature was associated with worse biochemical recurrence (BCR)-free survival in an independent cohort (*n* = 76 patients) [11]. Low: *n* = 49, 20 events; High: *n* = 26, 15 events. PGA: proportion of the genome with a copy number abberation; IDC/CA: intraductal carcinoma or cribriform architecture; mpMRI: multiparametric magnetic resonance imaging; FDR: false discovery rate; ρ: Spearman’s rho; FC: fold change; HR: hazard ratio

To identify protein-coding RNAs and proteins associated with mpMRI visibility and disease aggression, we next focused on the nimbosus hallmarks [3, 7] and small nucleolar RNAs (snoRNA) that are associated with mpMRI visibility [3, 7]. These hallmarks were previously shown to be associated with mpMRI visibility and disease aggression at the genomic and transcriptomic level [3]. An independent discovery cohort of 144 National Comprehensive Cancer Network (NCCN) intermediate-risk tumors was used to discover associations between RNA abundance and each hallmark (Additional file 1: Methods) [11, 12]. We identified 14,044 protein-coding RNAs and 1,622 proteins associated with at least one nimbosus hallmark in this cohort (Fig. 2A, Additional file 1: Methods). Proportion of the genome with a copy number aberration (PGA) and IDC/CA status showed the largest effects on the transcriptome and proteome. Proteins more abundant in mpMRI-invisible tumors were also negatively correlated with these hallmarks (Fig. 2B). Proteins associated with high PGA were preferentially associated with mpMRI visibility (hypergeometric test *p* = 3.3 × 10^–2^; Fig. 2C). mpMRI visibility was also strongly associated with aggressive hallmarks such as hypoxia, presence of IDC/CA, and *SChLAP1* expression through proteins, rather than protein-coding RNAs (Fig. 2D).

Finally, we employed a machine learning approach to find proteins that best differentiate mpMRI-visible and mpMRI-invisible tumors in our cohort. Following feature selection, we created a three-protein logistic regression model (LDHB, GNA11, SRD5A2) that classified mpMRI visibility status with an AUC of 0.88 (95% CI = 0.77–0.98, Fig. 2E, Additional file 1: Methods). This model was associated with worse biochemical recurrence-free survival in an independent cohort of 76 predominantly NCCN intermediate-risk tumors (HR = 1.79, 95% CI = 0.92–3.51, *p* = 0.089, median follow-up 6.02 years, Fig. 2E, inset) [11], further supporting the association between proteomic determinants of mpMRI visibility and tumor aggressiveness.

These data establish that mpMRI visibility is largely independent of the molecular features of tumor-adjacent stromal cells in the prostate. Rather, the proteome of mpMRI-invisible tumors is more similar to that of normal tissues [4, 10], suggesting that mpMRI visibility reflects the degree of proteomic dysregulation. Caveats of this study include uncertain generalization beyond ISUP Grade Group 2 tumors, the Caucasian ancestry of most patients, and study of only PI-RADSv2 scores of 1–2 and 5. These data suggest that tumors are invisible to mpMRI because their proteome does not differ sufficiently from normal prostate.

## Supplementary Information

**Additional file 1** **Methods.** Method details.

**Additional file 2.** **Fig. S1.** Tumor/NAT differences. **A** Consensus clustering of samples (n = 81, K = 4) using the top 25% most variable proteins (n = 1,193, K = 4). **B** Differentially abundant protein-coding RNAs in tumors and NATs from The Cancer Genome Atlas (TCGA). Statistically significant genes (FDR < 0.05) are colored in black. **C** Associations of protein abundance changes between tumor versus NAT, and mpMRI-visible NAT versus mpMRI-invisible NAT. Only proteins that were significantly differentially expressed in tumor and NAT regions (FDR < 0.05) were used for this analysis. **D** Associations of protein-coding RNA abundance changes between tumor versus NAT, and mpMRI-visible tumor versus mpMRI-invisible tumor. Only protein-coding RNAs that were significantly differentially expressed in tumor and NAT regions (FDR < 0.05) were used for this analysis. Proteins or transcripts that were significant (FDR < 0.05) in the tumor-NAT comparison and had the same directionality are marked in black. NAT: histologically normal prostate adjacent to the tumor; mpMRI: multiparametric magnetic resonance imaging; FDR: Benjamini-Hochberg-adjusted *p*-value.

**Additional file 3.** **Table S1.** Summary and characteristics of patient cohort.

**Additional file 4.** **Table S2. **Proteins quantified by mass spectrometry.

**Additional file 5.** **Table S3. **Pathway analysis of protein clusters.

## Data Availability

Mass spectrometry data and proteinGroups.txt output table was deposited in the MassIVE database under the accession MSV000088000 at ftp://massive.ucsd.edu/MSV000088000/. Oncoscan Copy Number Aberration (CNA) data and RNA-seq data can be found at the European Genome-phenome Archive (EGA) under accession EGAS00001003179.

## Abbreviations

95% CI: 95% Confidence interval
ARI: Adjusted Rand Index
AUC: Area under the curve
EMT: Epithelial-to-mesenchymal transition
IDC/CA: Intraductal carcinoma or cribriform architecture histology
ISUP: International Society of Urological Pathology
mpMRI: Multiparametric magnetic resonance imaging
NAT: Adjacent histologically normal tissue
NCCN: National Comprehensive Cancer Network
PGA: Proportion of the genome with a copy number aberration
PI-RADSv2: Prostate Imaging Reporting and Data System version 2
snoRNA: Small nucleolar RNA
