# Supplementary material for: Prostate cancer multiparametric magnetic resonance imaging visibility is a tumor-intrinsic phenomena
Source: J Hematol Oncol. 2022 May 3;15:48. doi: 10.1186/s13045-022-01268-6 (PMC9066728; doi:10.1186/s13045-022-01268-6)
Supplement: Supplementary file 1 — Additional file 1 Methods. Method details. [file 13045_2022_1268_MOESM1_ESM.docx]

# Supplementary Methods

## Patient Cohort and Tumor Sectioning

Patient selection, mpMRI acquisition and interpretation, tissue collection, and sample processing has been previously described(1). The study was approved by the University of California Los Angeles Institutional Research Ethics Board. Written informed consent was obtained from all patients. Tumor regions (n = 41) and normal tissue adjacent to the tumor (NAT) regions (n = 40) were annotated by a genitourinary pathologist. The relevant areas of tissue were macro-dissected from adjacent 10 µm sections for proteomics. For one patient, 2 tumor regions (Gleason 3 and Gleason 4) were macro-dissected separately, giving a total of 81 samples.

## Tissue preparation for shotgun proteomics

Each scraped FFPE tissue region was placed in a 1.5 mL conical tube and deparaffinized with xylene as follows. 500 µL of buffer was added to each tube, then samples were vortexed at high speed for 30 seconds, incubated for 5 minutes on an end-over-end nutator at room temperature, centrifuged at 18,000 rcf for 3 min, and the supernatant was discarded. The deparaffinization step was repeated twice. Tissues were then rehydrated with a graded ethanol series (95% ethanol, 90% ethanol, 75% ethanol, 50% ethanol, 25% ethanol, and water). Water was evaporated from each tube using a SpeedVac vacuum concentrator (Thermo) until 100 µL of water remained in each tube. 10 µL of 1M Tris pH 8.0 buffer was added to each sample to a final concentration of 100 mM Tris-HCl, pH 8. Samples were heated at 95°C for 1 hour to reverse formalin-induced crosslinking, then sonicated (Hielscher VialTweeter). Protein digestion was performed as previously described(2). Synthetic iRT peptides (Biognosis) were spiked into each sample at a 1:10 ratio prior to data acquisition. LC-MS/MS data was acquired using an Easy nLC 1000 (Thermo) nano-flow liquid chromatography system with a 50 cm EasySpray ES803 column (Thermo) coupled to a Q Exactive HF (Thermo) tandem mass spectrometer. Peptides were separated by reverse phase chromatography using a 4-hour chromatographic gradient. Mass spectrometry data was acquired in data dependent mode. Data was searched in MaxQuant (version 1.6.1.0) using a merged UniProt protein sequence database containing human and yeast invertase (Suc2) sequences, and iRT synthetic peptide sequences (Biognosis). LFQ intensities were used for protein quantitation. For proteins with missing LFQ values, median-adjusted iBAQ values were used as replacement(3). No batch correction was performed and no samples were excluded from analysis.

## Consensus clustering of proteomic data

Consensus clustering (ConsensusClusterPlus v1.52.0) was performed using divisive hierarchical clustering on the 25% most variable proteins in the cohort to cluster samples and proteins. Missing values were imputed with random values drawn from a normal distribution of protein abundances (width = 0.2; down-shift = 1.8)(4). Adjusted Rand Index (ARI) (CrossClustering v.4.0.3) was calculated between sample subtypes generated from consensus clustering and two other subtypes: Tumor and NAT, or samples from patients with mpMRI-visible versus mpMRI-invisible tumors.

## Differential abundance analysis

Mann-Whitney U-test was used for all comparisons. For each comparison, proteins present in >50% of the samples were kept for further analysis: visible versus invisible NAT (n_samples_ = 40, n_proteins_ = 4,165), visible versus invisible tumor (n_proteins_ = 4,426), tumor versus NAT (n_samples_ = 80, n_proteins_ = 4,314). Proteins in the intersection of these 3 sets (n_proteins_ = 4,067) were used for differential abundance analysis, with multiple testing correction using the Benjamini-Hochberg method. Missing values were imputed with random values drawn from a normal distribution of protein abundances (*width=*0.2; *down-shift*=1.8)(4).

For Tumor *vs.* NAT comparison of protein-coding RNAs, the tumor data used for this analysis were obtained from The Cancer Genome Atlas (TCGA) cBioPortal(9) on July 2020 (n_tumor_ = 499, n_NAT_ = 53). Mann-Whitney U-test was used for all comparisons (n_RNA_ = 20,233), with multiple testing correction using the Benjamini-Hochberg method. Missing values were imputed with random values drawn from a random uniform distribution of RNA transcript per million counts between 0 and 1.

## Similarity between groups

Euclidean distance was calculated between the protein abundance of each sample and median abundance of NAT. Only proteins detected in all samples (n = 2,309) were used. IDC/CA groups were determined based on the presence of intraductal carcinoma (IDC) or cribriform architecture (CA) pathology (IDC/CA+, n = 11) or not (IDC/CA-, n = 29). Hypoxia groups (n = 20 per group) were determined by median dichotomization (median score = -1) (5).

## Pathway enrichment analysis

Pre-ranked gene set enrichment analysis (GSEA)(6,7) (v.4.0.3) was performed to identify hallmark gene sets(8) that were enriched in each group. For each comparison, proteins were ranked by log_2_ fold change. GSEA was run on each group and adjusted for significance separately but visualized together to better show potential overlaps in hallmark gene sets.

## Association analysis of mpMRI visibility hallmarks with RNA and protein abundances

To identify protein-coding RNAs associated with mpMRI visibility hallmarks, univariate association tests - Spearman’s *ρ* for continuous values, Mann-Whitney U-test for binary values - were performed with each RNA (log_2_-transformed transcripts per million [TPM]) in the discovery cohort(2) (n = 144) against the following mpMRI visibility hallmarks(1): percent genome altered (PGA), hypoxia (Ragnum score), presence of intraductal carcinoma or cribriform architecture (IDC/CA), and expression of 8 non-coding RNAs (*SChLAP1, SNORA12, SNORA54, SNORD68, SNORD3A, SNORD33, SNORA37, SCARNA5*). RNAs that had <5 transcripts per million (TPM) in all samples or were detected in less than 2 samples were excluded from further analysis. RNAs that were associated with at least one hallmark in the discovery cohort (n_RNA_ = 15,368; FDR < 0.2) were evaluated in this cohort(1) (n_patient_ = 40). Hallmark-associated protein-coding RNAs that validated (n = 14,044) that had a corresponding protein detected (n = 3,791) in this cohort were carried forward for validation of protein associations with visibility hallmarks (n = 40). Proteins were considered validated if they were also significantly associated with the same hallmark at the protein level (n = 1,780; FDR < 0.2) and had the same directionality as the corresponding RNA association.

## Protein signature to predict mpMRI-visible tumors

We considered proteins detected in all tumor samples (n = 2,710) for mpMRI-visibility protein model development. For the one patient with two annotated tumor regions processed separately, tumor protein abundance for that patient was determined by taking the maximum protein abundance for each protein. Protein abundances were log_2_-transformed and selected for associations with mpMRI-visibility using leave-one-out (n = 40) cross validation (LOOCV) of Least Absolute Shrinkage and Selection Operator (LASSO) logistic regression (glmnet v4.1), with inner LOOCV for lambda selection (“one-standard-error”). The number of times each predictor was selected by the LASSO model was tallied across all folds and three proteins (LDHB, SRD5A2, GNA11) were consistently chosen as predictors (chosen in at least 5 out of 40 folds) and were used to build a logistic regression model to predict tumor visibility. The performance of the model was assessed using LOOCV (n = 40), with receiver operating characteristic (ROC) and area under the ROC curve (AUC) confidence intervals calculated using the pROC package (v1.17.0.1).

To further determine whether the model is associated with prostate cancer aggressiveness, we tested its associations with biochemical-relapse-free survival in an independent cohort, due to the lack of outcome data in our cohort. We first trained the model on the full mpMRI cohort (n = 40). The final logistic regression model had intercept of 274.388, and coefficients of (-)6.439, (-)1.824 and (-)1.807 for LDHB, SRD5A2, GNA11, respectively. We then applied it to an independent cohort of 76 intermediate-risk prostate cancer samples with log_2_-transformed protein abundance(2). In this independent cohort, samples were dichotomized by model output at the cutoff of 0.5 and the sample groups were tested for differences in biochemical-relapse-free survival using Cox proportional-hazards modeling. Model performance for mpMRI-visibility prediction was not assessed in the independent cohort since imaging data was not available.

# Supplementary References

1. Houlahan KE, Salmasi A, Sadun TY, Pooli A, Felker ER, Livingstone J, et al. Molecular Hallmarks of Multiparametric Magnetic Resonance Imaging Visibility in Prostate Cancer. Eur Urol. 2019 Jul 1;76(1):18–23.

2. Sinha A, Huang V, Livingstone J, Wang J, Fox NS, Kurganovs N, et al. The Proteogenomic Landscape of Curable Prostate Cancer. Cancer Cell. 2019 Mar 18;35(3):414-427.e6.

3. Wojtowicz EE, Lechman ER, Hermans KG, Schoof EM, Wienholds E, Isserlin R, et al. Ectopic miR-125a Expression Induces Long-Term Repopulating Stem Cell Capacity in Mouse and Human Hematopoietic Progenitors. Cell Stem Cell. 2016;19(3):383–96.

4. Tyanova S, Temu T, Sinitcyn P, Carlson A, Hein MY, Geiger T, et al. The Perseus computational platform for comprehensive analysis of (prote)omics data. Nat Methods. 2016 Sep;13(9):731–40.

5. Ragnum HB, Vlatkovic L, Lie AK, Axcrona K, Julin CH, Frikstad KM, et al. The tumour hypoxia marker pimonidazole reflects a transcriptional programme associated with aggressive prostate cancer. Br J Cancer. 2015 Jan;112(2):382–90.

6. Subramanian A, Tamayo P, Mootha VK, Mukherjee S, Ebert BL, Gillette MA, et al. Gene set enrichment analysis: a knowledge-based approach for interpreting genome-wide expression profiles. Proc Natl Acad Sci U S A. 2005 Oct;102(43):15545–50.

7. Mootha VK, Lindgren CM, Eriksson K-F, Subramanian A, Sihag S, Lehar J, et al. PGC-1alpha-responsive genes involved in oxidative phosphorylation are coordinately downregulated in human diabetes. Nat Genet. 2003 Jul;34(3):267–73.

8. Liberzon A, Birger C, Thorvaldsdóttir H, Ghandi M, Mesirov JP, Tamayo P. The Molecular Signatures Database (MSigDB) hallmark gene set collection. Cell Syst. 2015 Dec;1(6):417–25.

9. Cerami et al. The cBio Cancer Genomics Portal: An Open Platform for Exploring Multidimensional Cancer Genomics Data. Cancer Discovery. May 2012 2; 401. PubMed.
