# Supplementary figures and images for: Prostate cancer multiparametric magnetic resonance imaging visibility is a tumor-intrinsic phenomena
Source: J Hematol Oncol. 2022 May 3;15:48. doi: 10.1186/s13045-022-01268-6 (PMC9066728; doi:10.1186/s13045-022-01268-6)

# Supplementary Figure 1

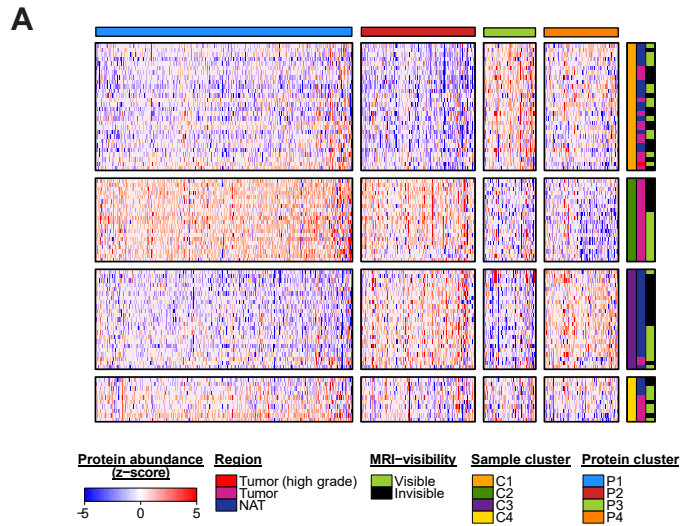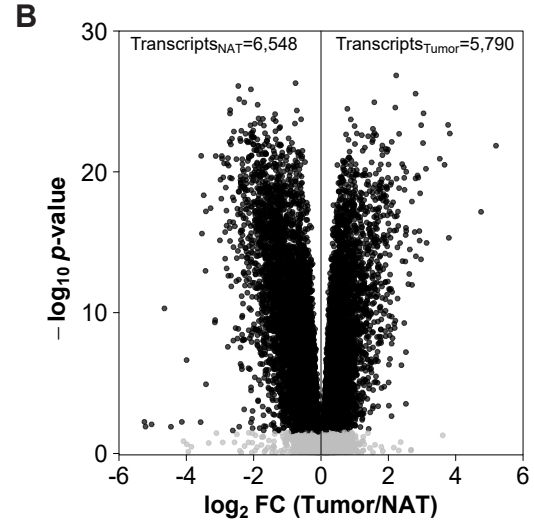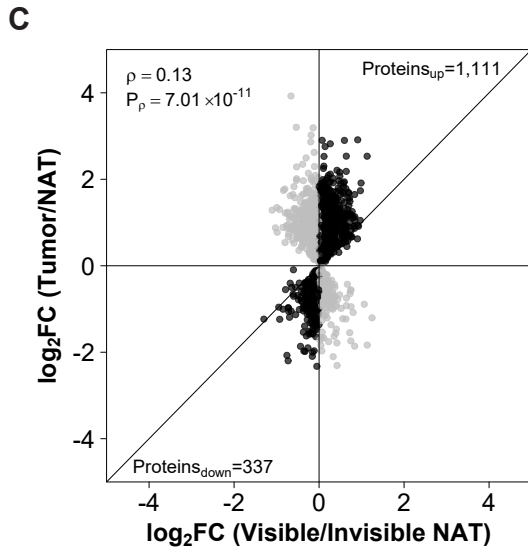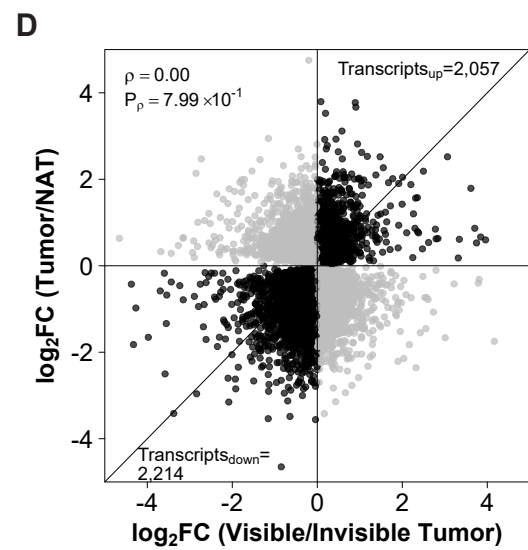

Supplement: Supplementary file 2 — Additional file 2. Fig. S1. Tumor/NAT differences. A Consensus clustering of samples (n = 81, K = 4) using the top 25% most variable proteins (n = 1,193, K = 4). B Differentially abundant protein-coding RNAs in tumors and NATs from The Cancer Genome Atlas (TCGA). Statistically significant genes (FDR < 0.05) are colored in black. C Associations of protein abundance changes between tumor versus NAT, and mpMRI-visible NAT versus mpMRI-invisible NAT. Only proteins that were significantly differentially expressed in tumor and NAT regions (FDR < 0.05) were used for this analysis. D Associations of protein-coding RNA abundance changes between tumor versus NAT, and mpMRI-visible tumor versus mpMRI-invisible tumor. Only protein-coding RNAs that were significantly differentially expressed in tumor and NAT regions (FDR < 0.05) were used for this analysis. Proteins or transcripts that were significant (FDR < 0.05) in the tumor-NAT comparison and had the same directionality are marked in black. NAT: histologically normal prostate adjacent to the tumor; mpMRI: multiparametric magnetic resonance imaging; FDR: Benjamini-Hochberg-adjusted p-value. [file 13045_2022_1268_MOESM2_ESM.pdf]
